# ATIC inhibits autophagy in hepatocellular cancer through the AKT/FOXO3 pathway and serves as a prognostic signature for modeling patient survival

**DOI:** 10.7150/ijbs.65669

**Published:** 2021-10-25

**Authors:** Hao Zhang, Peng Xia, Jie Liu, Zhang Chen, Weijie Ma, Yufeng Yuan

**Affiliations:** 1 Department of Hepatobiliary and Pancreatic Surgery, Zhongnan Hospital of Wuhan University, Donghu Road 169#, Wuhan 430071, China.; 2 Clinical Medicine Research Center for Minimally Invasive Procedure of Hepatobiliary & Pancreatic Diseases of Hubei Province, Hubei, China.

**Keywords:** hepatocellular carcinoma, autophagy-related genes, prognosis, immune cell infiltration, ATIC

## Abstract

**Background:** Autophagy regulates many cell functions related to cancer, ranging from cell proliferation and angiogenesis to metabolism. Due to the close relationship between autophagy and tumors, we investigated the predictive value of autophagy-related genes.

**Methods:** Data from patients with hepatocellular carcinoma were obtained from The Cancer Genome Atlas (TCGA) and the International Cancer Genome Consortium (ICGC) databases. A regression analysis of differentially expressed genes was performed. Based on a prognostic model, patients were divided into a high-risk or low-risk group. Kaplan-Meier survival analyses of patients were conducted. The immune landscapes, as determined using single-sample gene set enrichment analysis (ssGSEA), exhibited different patterns in the two groups. The prognostic model was verified using the ICGC database and clinical data from patients collected at Zhongnan Hospital. Based on the results of multivariate Cox regression analysis, 5-aminoimidazole-4-carboxamide ribonucleotide formyltransferase/inosine monophosphate (IMP) cyclohydrolase (ATIC) had the largest hazard ratio, and thus we studied the effect of ATIC on autophagy and tumor progression by performing *in vitro* and *in vivo* experiments.

**Results:** Fifty-eight autophagy-related genes were differentially expressed (false discovery rate (FDR)<0.05, log_2_ fold change (logFC)>1); 23 genes were related to the prognosis of patients. A prognostic model based on 12 genes (ATG10, ATIC, BIRC5, CAPN10, FKBP1A, GAPDH, HDAC1, PRKCD, RHEB, SPNS1, SQSTM1 and TMEM74) was constructed. A significant difference in survival rate was observed between the high-risk group and low-risk group distinguished by the model (*P*<0.001). The model had good predictive power (area under the curve (AUC)>0.7). Risk-related genes were related to the terms type II IFN response, MHC class I (*P*<0.001) and HLA (*P*<0.05). ATIC was confirmed to inhibit autophagy and promote the proliferation, invasion and metastasis of liver cancer cells through the AKT/Forkhead box subgroup O3 (FOXO3) signaling pathway *in vitro* and *in vivo*.

**Conclusions:** The prediction model effectively predicts the survival time of patients with liver cancer. The risk score reflects the immune cell features and immune status of patients. ATIC inhibits autophagy and promotes the progression of liver cancer through the AKT/FOXO3 signaling pathway.

## Introduction

Worldwide, liver cancer is responsible for the 4^th^ highest number of cancer-related deaths, and the number of new liver cancer cases diagnosed annually is the 6^th^ highest among all cancers 1. The 5-year survival rate of patients with liver cancer is only 18%, and it is the second most lethal cancer after pancreatic cancer. Furthermore, the long-term survival rate of patients with liver cancer varies substantially among countries and regions 2, 3. The molecular characteristics of liver cancer cells and the clinical prognosis of liver cancer are very different from those of other cancers, which leads to challenges in predicting the prognosis of patients with liver cancer 4, 5.

Autophagy increases the viability of tumor cells and exacerbates tumor progression 6. Numerous studies have reported a connection between autophagy and the human immune system and immune-related functions. Recent studies have reported that autophagy reduces cell surface inhibition of antitumor immune responses and promotes immune evasion in pancreatic cancer 7. Based on the close relationship between autophagy and immunity, we analyzed autophagy and immune-related functions in this study. In summary, autophagy plays an important role in tumorigenesis and tumor progression, and immunotherapy will be an important strategy for tumor treatment in the future 8.

Due to the close relationship between autophagy and tumors, we designed this study to explore the relationship between autophagy and the prognosis of patients with liver cancer. Here, we identified genes that are closely related to the prognosis of patients with liver cancer using bioinformatics methods such as Cox regression analysis and survival analysis, provided ideas for subsequent mechanistic studies, and established models for predicting survival.

The results of multivariate Cox regression analysis revealed the largest hazard ratio for 5-aminoimidazole-4-carboxamide ribonucleotide formyltransferase/inosine monophosphate (IMP) cyclohydrolase (ATIC), suggesting that ATIC is closely related to patient prognosis. Therefore, we chose ATIC for further verification. ATIC is considered related to autophagy, but experimental evidence is not available to prove the relationship between ATIC and autophagy 9. In the present study, we determined the relationship between ATIC and tumor cell autophagy by performing *in vivo* and *in vitro* experiments. We found that ATIC inhibited autophagy and promoted liver cancer progression by modulating the AKT/Forkhead box subgroup O3 (FOXO3) pathway.

This article identified autophagy-related genes related to the prognosis of liver cancer, analyzed the relationship of prognosis-related autophagy genes with immune cells and immune-related functions, and constructed a model to predict the survival of patients with liver cancer in the clinic using bioinformatics methods. Furthermore, we have suggested that ATIC inhibits autophagy and promotes liver cancer progression through the AKT/FOXO3 pathway.

## Materials and Methods

### Data collection

The mRNA sequencing data of 50 normal samples and 374 liver cancer samples and clinical data from 374 patients with liver cancer were downloaded from The Cancer Genome Atlas (TCGA) 10. Then, mRNA sequencing data from 240 patients with liver cancer were downloaded from the International Cancer Genome Consortium (ICGC) 11. After excluding patients with incomplete data, 365 patients from TCGA database and 231 patients from the ICGC database were included in the analysis. A list of 256 autophagy-related genes was obtained from the Human Autophagy Database 12. The expression levels of autophagy-related genes were extracted from TCGA sequencing data.

### Identification of differentially expressed genes and prognosis-related genes

The “limma” R package was used to perform the differential expression analysis. The screening conditions were a log2 fold change (logFC)>1 and false discovery rate (FDR)<0.05. The survival R package was used to identify prognosis-related genes. The genes that were both differentially expressed and related to prognosis were determined. A protein interaction network was generated for the overlapping genes. The “igraph” and “reshape2” R packages were used to calculate the correlations between the expression levels of overlapping genes and to determine the correlation network (correlation threshold: 0.4).

### Construction of prognostic models

We used both LASSO regression and multivariate Cox regression analyses to establish prognostic models based on TCGA data. Then, we used ICGC data to verify the established models. According to the model, ICGC samples were divided into a high-risk and low-risk group. The predictive power of the models was evaluated by performing a survival analysis, ROC curve analysis and independent prognostic analysis. The "Rtsne" R package was used for principal component analysis (PCA), and t-stochastic neighbor embedding (t-SNE) was used for dimensionality reduction and data visualization. After the two models were tested and compared, the prognostic model established using the LASSO regression method had better predictive power. Therefore, we used the prognostic model established using the LASSO regression method for subsequent analyses.

### Identification of risk-related genes, gene set enrichment analysis and immune cell infiltration analysis

The “limma” R package was used to analyze the differentially expressed genes in the high-risk and low-risk groups. Then, GO functional enrichment analysis and Kyoto Encyclopedia of Genes and Genomes (KEGG) pathway enrichment analysis were performed with the risk-related genes obtained from the analysis of TCGA and ICGC data. Gene set enrichment analysis was performed using the “clusterProfiler” and “org.Hs.eg.db” R packages, and a P value<0.05 and q value<0.05 were used as thresholds.

According to a previous study, gene expression levels in tumors are related to local immune cell activity and immune-related functions 13. Therefore, we used the single-sample gene set enrichment analysis (ssGSEA) function in the “GSVA” R package to calculate the immune-related scores for each sample and the relationship between the immune-related scores and risk scores. We used the same method to calculate the relationship between immune cell infiltration and the expression of the representative gene ATIC in the model.

### Cell culture and transfection

Huh7, HepG2 and HCCLM3 liver cancer cells were obtained from Shanghai Cell Bank (Shanghai, China). The cell lines had recently been authenticated and tested for mycoplasma. Huh7, HepG2 and HCCLM3 cells were grown in DMEM (Invitrogen, Carlsbad, CA). All media were supplemented with 10% fetal bovine serum (Invitrogen). Jtsbio (Guangzhou, China) constructed and synthesized the siRNA and shRNA lentivirus vector specific for ATIC. The sequence of the shRNA is the same as siRNA 3:

ATIC-siRNA 1: forward, 5'-CCUGCAAUCUCUAUCCCUUTT -3'; reverse, 5'-AAGGGAUAGAGAUUGCAGGTT -3' and;

ATIC-siRNA 3: forward, 5'-GGUGUCGUCGACAAGUCAUTT -3'; reverse, 5'-AUGACUUGUCGACGACACCTT -3'.

For ATIC overexpression, the full-length ATIC cDNA was amplified by PCR and cloned into the expression vector pcDNA3.1 (Invitrogen). The empty vector was used as a control. Transfection was performed using Lipofectamine 3000 reagent (Invitrogen). Forty-eight hours after transfection, cells were harvested for further analysis.

### RNA extraction and RT-qPCR analysis

Total RNA was extracted from the prepared tissues and cells using TRIzol reagent (Invitrogen). Complementary DNA was synthesized using a PrimeScript RT kit (TaKaRa, Dalian, China). Then, SYBR Green Master Mix (Applied Biosystems, Foster City, CA, USA) was used to perform RT-qPCR with the ABI PRISM 7900 Sequence Detection System (Applied Biosystems). The data were calculated using the 2-ΔΔCt method, and GAPDH was used as an endogenous control. The primer sequences are as follows:

ATIC-F: 5'- CACGCTCGAGTGACAGTG- 3'; ATIC-R: 5'- TCGGAGCTCTGCATCTCCG- 3';

GAPDH-F: 5'-GTCTCCTCTGACTTCAACAGCG -3'; and GAPDH-R: 5'- ACCACCCTGTTGCTGTAGCCAA-3'.

### Transmission electron microscopy

Conventional electron microscopy was performed as described below. Cells were fixed with 2.5% glutaraldehyde and then postfixed with 1% osmium tetroxide. The samples were dehydrated in a series of ethanol solutions and embedded in Embed812 resin. Ultrathin sections were cut with a diamond knife. Next, the sections were mounted on copper grids and double-stained with uranyl acetate and lead citrate. The number of autophagic vacuoles was determined for a minimum of 100 cells.

### Detection of cell viability and migration

Cell Counting Kit 8 (CCK-8) was used to measure cell viability. An Infinite M200 spectrophotometer (Tecan) was used to detect the absorbance of each sample at 450 nm. Transwell assays were conducted to assess cell migration. BioCoat Matrigel Invasion Chambers (BD Biosciences) were used to assess cell invasion. Three random areas were assessed to determine the cell counts in the cell migration and invasion experiments. An annexin V-FITC apoptosis detection kit (Invitrogen) was used for annexin V and propidium iodide staining of cells, and the percentage of apoptotic cells was determined using flow cytometry (Beckman, USA). Forty-eight hours after transfection, the cells were stained with propidium iodide and assessed using fluorescence-activated cell sorting (FACS) to assess the cell cycle. LY294002 (T2008), MK2206 (T1952) and SC79 (T2274) were purchased from Topscience (Shanghai, China).

### Colony formation assay

The treated liver cancer cells were cultured in a 6-well plate. After 14 days, the culture medium was discarded, and the cells were washed twice with PBS. Then, paraformaldehyde was used to fix the cells at room temperature for 20 minutes, 0.1 mM crystal violet was added, the cells were stained for 10 minutes, washed twice with PBS, and the results were analyzed under a microscope.

### Western blot analysis

The anti-p-PI3K antibody (ab182651), anti-PI3K antibody (ab140307), anti-AKT antibody (ab8805), anti-p-AKT antibody (ab38449), anti-LC3 antibody (ab192890) and anti-GAPDH antibody (ab8245) were purchased from Abcam. Antibodies against caspase 3 (19677-1-AP), caspase 7 (27155-1-AP), caspase 9 (10380-1-AP), FOXO1 (66457-1-Ig), FOXO3 (66428-1-Ig), and ATIC (10726-1-AP) and secondary antibodies (SA00001-1 and SA00001-2) were purchased from Proteintech (Wuhan, China). Cell samples were lysed in sodium dodecyl sulfate (SDS, Beyotime, Shanghai, China). The protein concentration was determined with the Enhanced BCA Protein Assay Kit (Beyotime), and proteins were separated by SDS-polyacrylamide gel electrophoresis and transferred to a polyvinylidene fluoride (PVDF) membrane (Millipore, Bedford, MA, USA). After blocking with 5% skim milk for 1 hour, membranes were incubated with a specific primary antibody overnight at 4 °C and then incubated with the secondary antibody at room temperature for 2 hours. Protein bands were detected using an enhanced chemiluminescence (ECL) system (Pierce Biotech, Rockford, Illinois, USA). An anti-GAPDH antibody (1:1,000, Sigma, St. Louis, Missouri, USA) was used to detect GAPDH as a loading control.

### Xenotransplantation and lung metastasis models

For these experiments, 6-week-old BALB/c nude mice were used. The treated liver cancer cells were washed and suspended, and a single-cell suspension of 1×10^7^ cells/0.1 ml was inoculated into the armpits of nude mice. The long diameter (a) and short diameter (b) of subcutaneous xenograft tumors were measured every 5 days. The tumor volume was calculated using the formula V = ab ^2^/2. After 5 weeks, the nude mice were sacrificed, and the tumors were extracted. In another experiment, differently treated cells in the logarithmic growth phase were selected, and a cell suspension was prepared and injected through the tail vein. The nude mice were sacrificed after one month, and the lung tissues were collected for hematoxylin-eosin (H&E) staining. Generally, 1-5*10^6^ cells/200 µl were used. The animal studies were approved by the Animal Research Committee of Wuhan University.

### Statistical analysis

All statistical analyses were performed using GraphPad Prism 8.0 software (GraphPad Software, San Diego, California, USA) and SPSS 24.0 software (SPSS Inc., Chicago, Illinois, USA). Each experiment included at least three independent experiments. The continuous variable data are presented as the means ± standard deviations (SD). Statistical analysis was performed using unpaired Student's t-test to compare the continuous variables. To compare the categorical variables, χ^2^ test was performed to assess the pathological and clinical characteristics of the ATIC high/low groups. The differences between the experimental groups were assessed using Student's t-test or one-way ANOVA. Survival time between groups was evaluated using Kaplan-Meier method or univariate Cox regression analysis. A two-sided P value was calculated, and a probability level of 0.05 was considered statistically significant. ∗P < 0.05; ∗∗P< 0.01; ***p < 0.001.

## Results

### Identification of differentially expressed genes related to autophagy and the prognosis

**Figure 1:**
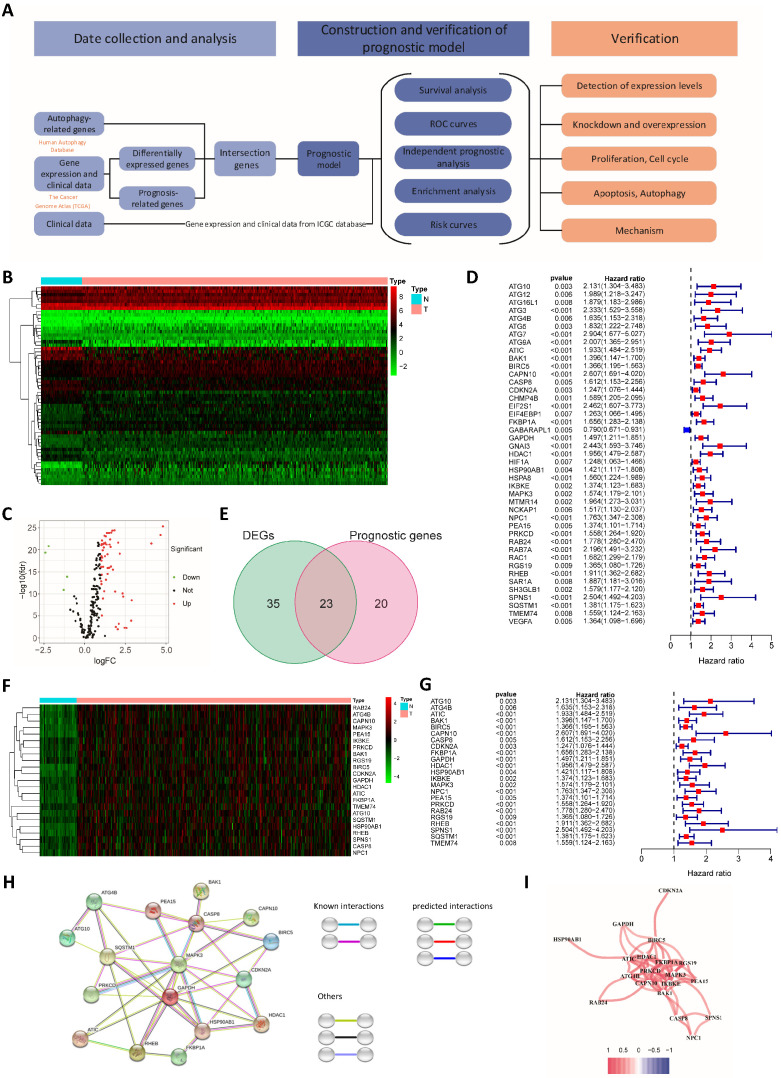
** Differentially expressed and prognosis-related genes.** (**A**) Flow chart. Heat map (**B**) and volcano plot (**C**) of differentially expressed genes (DEGs). (**D**) Prognosis-related genes. (**E**) Venn diagram. (**F**) Heat map of overlapping genes. (**G**) Hazard ratios of overlapping genes. (**H**) Protein interaction network. (**I**) Protein coexpression network. Red, high expression; green, low expression.

To better show the experimental process, we establish a flow chart (Fig. 1A). We first identified 58 genes that were abnormally expressed in tumors compared with normal tissues, of which 4 genes exhibited abnormally low expression and 54 genes displayed abnormally high expression (logFC>1 and FDR<0.05) (Fig. 1B and C). Forty-three prognosis-related genes were screened using a univariate Cox regression analysis (Fig. 1D). The intersection of the two gene sets resulted in 23 overlapping genes (Fig. 1E and Table S1).

The overlapping genes were all expressed at high levels in tumor tissues (Fig. 1F), and they were all factors contributing to an unfavorable prognosis (Fig. 1G). We generated a protein interaction network to explore the interactions and coexpression relationships between the overlapping genes. MAPK3, SQSTM1, CASP8 and HSP90AB1 were the core genes in the network and showed research potential (Fig. 1H). In the coexpression network, the lines between genes represent coexpression relationships, and red represents a positive correlation. IKBKE and PRKCD had the strongest coexpression relationship (Fig. 1I).

### The established model has satisfactory predictive power

**Figure 2:**
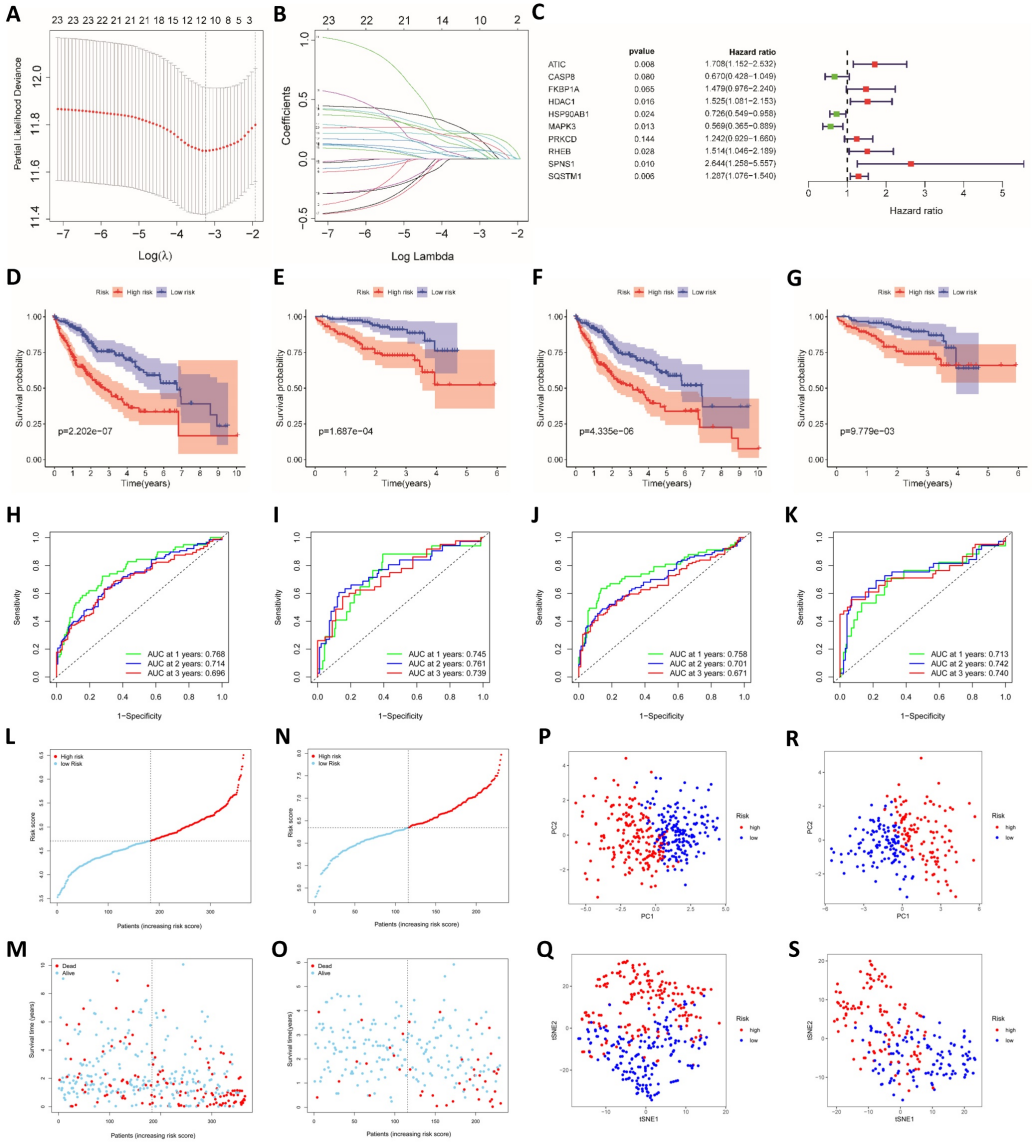
** Construction and verification of the prognostic models.** (**A**) Cross validation plot for the penalty term in the LASSO regression analysis. (**B**) LASSO regression coefficients for different values of the penalty parameter. (**C**) Hazard ratios of genes in the multivariate Cox regression analysis. Survival curves for the high-risk group and the low-risk group of patients in TCGA (**D**) and ICGC cohorts (**E**) based on the LASSO regression model. Survival curves for TCGA (**F**) and ICGC cohorts (**G**) based on the multivariate Cox regression model. ROC curves of TCGA (**H**) and ICGC cohorts (**I**) categorized with the LASSO regression model. ROC curves of TCGA (**J**) and ICGC cohorts (**K**) categorized with the multivariate Cox regression model. Distribution of risk scores (**L and N**) and survival statuses (**M and O**) in TCGA (L and M) and ICGC cohorts (**N and O**). PCA (**P**) and t-SNE analysis (**Q**) of patients in TCGA cohort. PCA (**R**) and t-SNE analysis (**S**) of patients in the ICGC cohort.

We used LASSO regression analysis and multivariate Cox regression analysis to construct prognostic models. The LASSO regression analysis identified 12 genes (ATG10, ATIC, BIRC5, CAPN10, FKBP1A, GAPDH, HDAC1, PRKCD, RHEB, SPNS1, SQSTM1 and TMEM74) (Fig. 2A and B). Based on these 12 genes, a prognostic model was constructed to predict patient survival. The risk score was calculated by multiplying the expression of each gene in the model by the corresponding coefficient and then adding the sum. The following formula was used to calculate the risk score obtained from the LASSO regression analysis:

Risk score = EX_ATG10_*0.265 + EX_ATIC_*0.216 + EX_BIRC5_*0.065 + EX_CAPN10_*0.034 + EX_FKBP1A_*0.114 + EX_GAPDH_*0.019 + EX_HDAC1_*0.230 + EX_PRKCD_*0.018 + EX_RHEB_*0.185 + EX_SPNS1_*0.189 + EX_SQSTM1_*0.094 + EX_TMEM74_*0.147 (EX, expression).

The prognostic model established based on the multivariate Cox regression analysis included 10 genes. ATIC, HDAC1, HSP90AB1, MAPK3, RHEB, SPNS1 and SQSTM1 were independent factors affecting the prognosis (Fig. 2C). The following formula was used to calculate the risk score from this model:

Risk score = EX_ATIC_*0.535 + EXCASP8*-0.400 + EX_FKBP1A_*0.391 + EX_HDAC1_*0.422 + EX_HSP90AB1_*-0.321 + EX_MAPK3_*-0.563 + EX_PRKCD_*0.216 + EX_RHEB_*0.415 + EX_SPNS1_*0.972 + EX_SQSTM1_*0.252.

We used data from the ICGC database to verify the model established based on TCGA data. Patients in the TCGA group and the ICGC group were divided into a high-risk group and a low-risk group according to the risk score. The risk score was related to the patient's disease stage and grade (Table 1). Furthermore, the difference in the prognosis of the two groups was calculated. The difference in survival between the high-risk group and the low-risk group was more obvious for the model established using the LASSO regression analysis than for the model established using the Cox regression analysis (Fig. 2D-G).

ROC risk curves were drawn, and the area under the curve (AUC) values were calculated to evaluate the predictive performance of the model. For the LASSO regression model, the 1-, 2-, and 3-year AUC values for TCGA patients were 0.768, 0.714 and 0.696, respectively (Fig. 2H). The ICGC group was used to test the efficacy of the model, and the AUC values were 0.745, 0.761 and 0.739, respectively (Fig. 2I). The AUC values of the model established based on the LASSO regression analysis were slightly lower than those of the model established based on the multivariate Cox regression (Fig. 2J and K). Therefore, the model established based on the LASSO regression analysis had better predictive performance, and the model was further verified and analyzed. In addition, we found that the hazard ratio calculated through the multivariate COX and the univariate COX regression analysis are opposite, such as MAPK3. We believe that this contradiction is due to Simpson's Paradox, which was proposed by British statistician E.H. Simpson in 1951. It refers. Therefore, we gave up the model established by multivariate COX regression analysis, and used the model established by LASSO regression for the further analysis. The distribution of risk scores among patients in TCGA cohort was symmetrical (Fig. 2L). The distribution of patient survival statuses showed that the survival time of patients in the high-risk group was shorter than that of patients in the low-risk group (P<0.01, Fig. 2M). When ICGC patient data were used for verification, the same distribution was observed (Fig. 2N and O). We conducted PCA and t-SNE analysis to show the clustering of risk scores more clearly. In both TCGA and ICGC groups, patients in the high-risk and low-risk groups were clearly distributed in different clusters (Fig. 2P-S).

### The risk score is an independent prognostic factor and is related to immune cells and immune function

**Figure 3:**
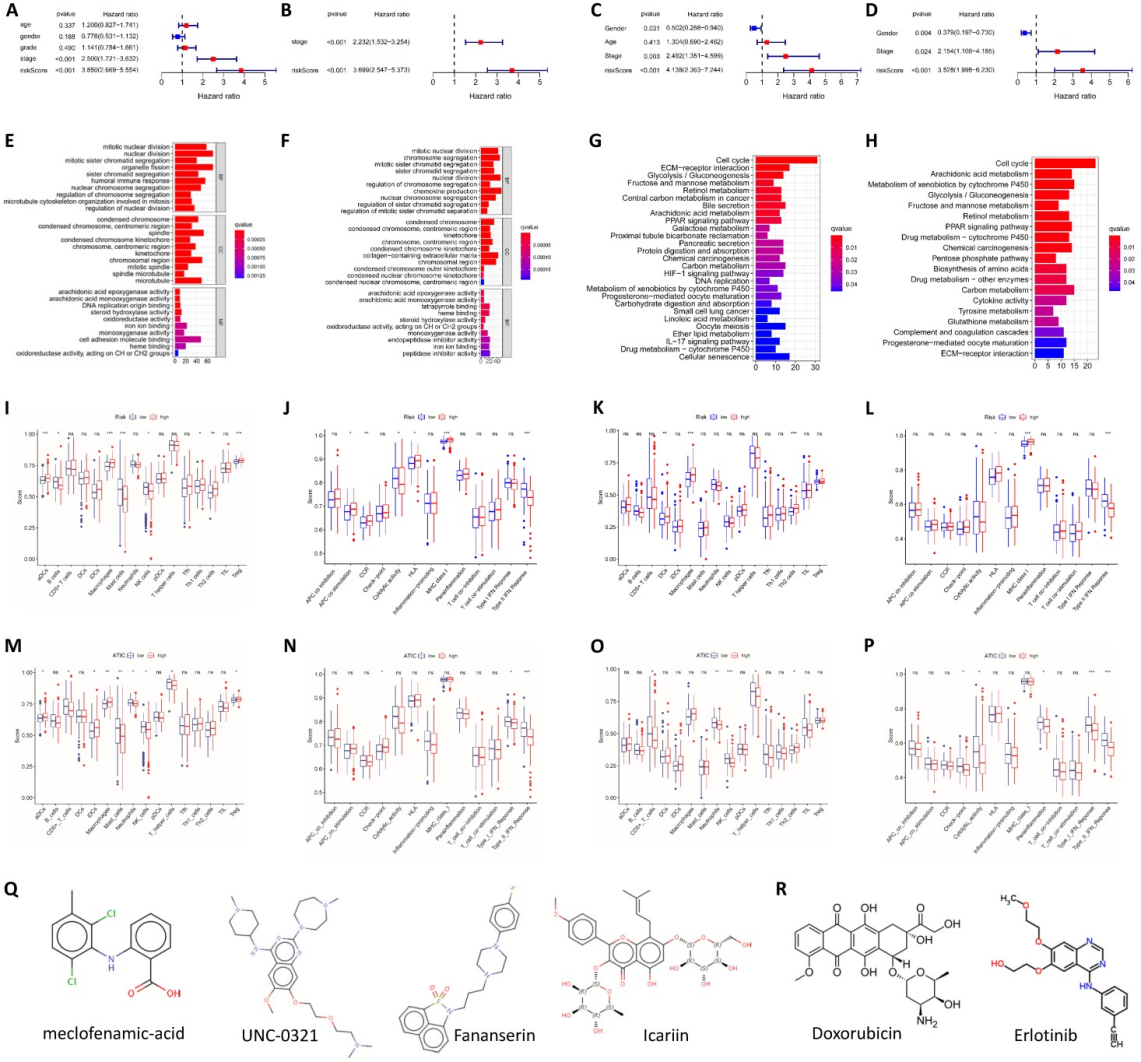
** Analysis of the independent prognostic value of the risk score and its association with immune cell infiltration.** (**A**) Univariate Cox regression analysis of TCGA cohort. (**B**) Multivariate Cox regression analysis of TCGA cohort. (**C**) Univariate Cox regression analysis of the ICGC cohort. (**D**) Multivariate Cox regression analysis of the ICGC cohort. GO functional enrichment analysis of TCGA (**E**) and ICGC cohorts (**F**). KEGG pathway enrichment analysis of TCGA (**G**) and ICGC cohorts (**H**). (**I**) The relationship between immune cell infiltration and risk score in TCGA cohort. (**J**) The relationship between immune function and risk score in TCGA cohort. (**K**) The relationship between immune cell infiltration and risk score in the ICGC cohort. (**L**) The relationship between immune function and risk score in the ICGC cohort. (**M**) The relationship between immune cell infiltration and ATIC expression in TCGA cohort. (**N**) The relationship between immune function and ATIC expression in TCGA cohort. (**O**) The relationship between immune cell infiltration and ATIC expression in the ICGC cohort. (**P**) The relationship between immune function and ATIC expression in the ICGC cohort. (**Q**) Candidate small-molecule compounds with therapeutic potential. (**R**) Drugs for which high-risk patients may show high resistance. ns, not significant.

We performed univariate and multivariate Cox regression analyses to determine whether the risk score was an independent prognostic indicator. In TCGA and ICGC groups, the disease stage and risk score were independent factors affecting the prognosis (P<0.001, Fig. 3A-D). We further performed an enrichment analysis of differentially expressed genes (logFC>1, adjusted P<0.05). The results of the GO analysis showed that the genes were mainly involved in cell cycle-related functions, including nuclear division and chromatic segregation (Fig. 3E and F). The results of the KEGG pathway enrichment analysis showed that autophagy-related genes were closely related to the cell cycle (Fig. 3G and H). In addition, autophagy-related genes were also related to immune function in the GO analysis and KEGG analysis. Related GO terms included humoral immune response, chemokine production, IL-17 signaling pathway and cytokine activity.

The differences in immune cells and functions were quantified and analyzed. Significant differences in the degree of activation of immune cells related to cellular immunity, including macrophages, natural killer (NK) cells, T helper 1 (Th1) cells, Th2 cells and regulatory T (Treg) cells, were observed between the high-risk group and low-risk group in TCGA cohort. In terms of immune-related functions, CCR and the type II IFN response, which are also related to cellular immunity, were significantly differentially activated between the high-risk and low-risk groups. In addition, immune terms related to antigen presentation, such as APC costimulation and MHC class I, were also significantly differentially enriched between the two groups (Fig. 3I and J). In the ICGC cohort, the degree of activation of macrophages and Th2 cells was obviously different between the high-risk and low-risk groups, similar to the result from TCGA. The changes in immune function in the ICGC group were the same as those in TCGA data, and a significant enrichment of MHC class I and the type II IFN response was observed in both cohorts (Fig. 3K and L).

For the key gene ATIC in the model, we divided patients into a high expression group and a low expression group according to the expression level of ATIC. In TCGA cohort, ATIC expression was related to the infiltration of CD8+ T cells, macrophages and mast cells (Fig. 3M). ATIC was related to immune checkpoints and type I and type II IFN response immune functions (Fig. 3N). In the ICGC cohort, ATIC was still associated with CD8+ T cells and NK cells, but not with macrophages (Fig. 3O). In the ICGC cohort, ATIC was related to immune checkpoints and the IFN response, consistent with the results obtained from TCGA data (Fig. 3P).

Based on the differentially expressed genes between the high-risk group and the low-risk group, we generated a connectivity map to identify small molecules with therapeutic potential using the online tool cMAP (Table S2). Among them, meclofenamic acid, UNC-0321, fananserin and icariin showed great therapeutic potential. Doxorubicin and erlotinib are used to treat liver cancer. For high-risk patients, these two drugs may exert a better effect (Fig. 3Q and R).

### ATIC is expressed at high levels in liver cancer tissues

**Figure 4:**
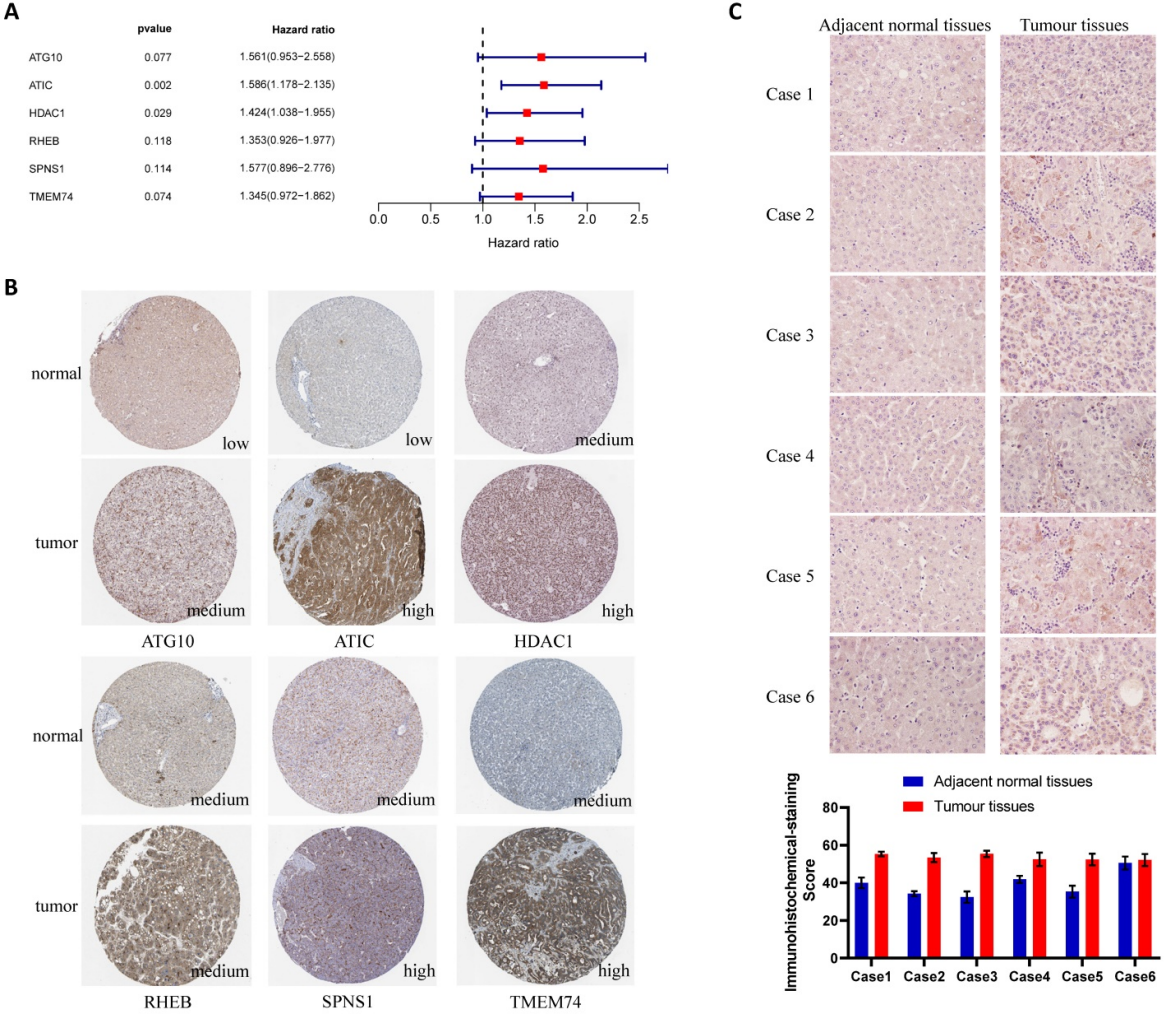
** Verification of the differential expression of risk-related genes in tissues and cells.** (**A**) Multivariate Cox regression analysis of genes in the LASSO regression model. (**B**) Expression of risk-related genes in liver cancer tissues and adjacent normal tissues (HPA database). (**C**) ATIC immunohistochemistry and quantitative results for patients from Zhongnan Hospital.

The results of the multivariate Cox regression analysis showed that ATIC and HDAC1 exerted significant effects on the prognosis (P<0.05, hazard ratio>1) (Fig. 4A). The immunohistochemical staining results were downloaded from the Human Protein Atlas (www.proteinatlas.org) and used according to their data usage policy 14. The expression of ATIC, HDAC1, RHEB, SPNS1 and TMEM74 in liver cancer tissues was significantly higher than that in normal liver tissues (Fig. 4B). Immunohistochemistry indicated that ATIC was expressed at high levels in tumor tissues but weakly expressed in adjacent normal tissues (Fig. 4C).

### High ATIC expression is associated with a poor prognosis

**Figure 5:**
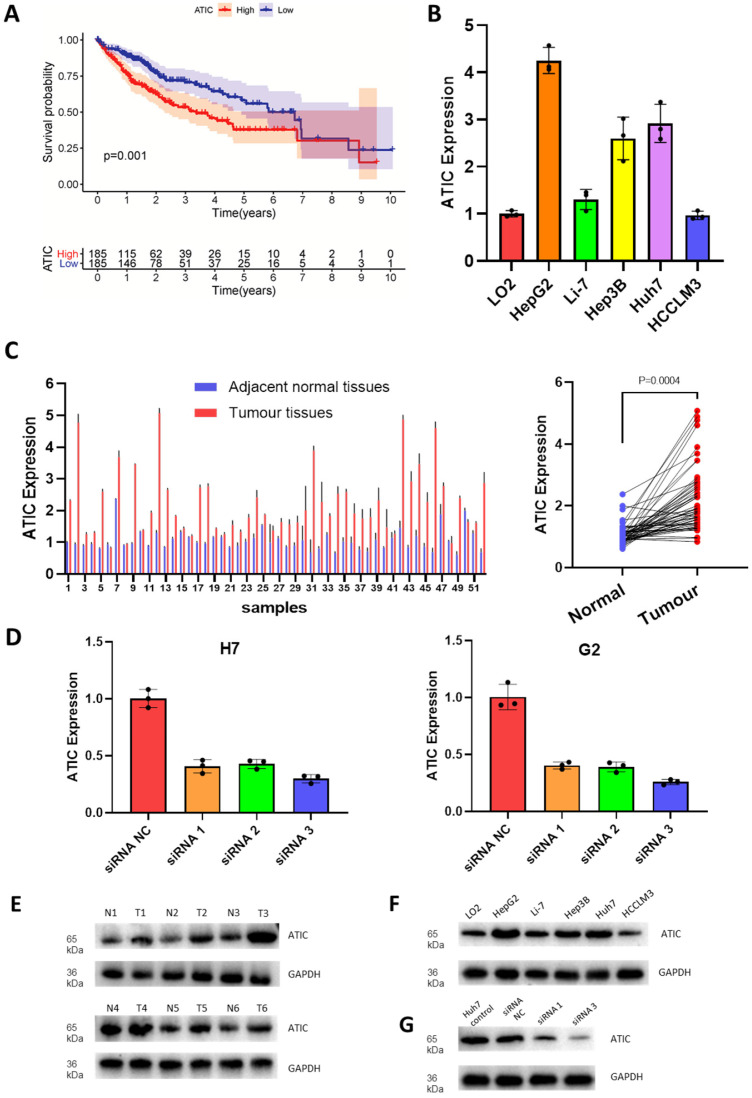
** ATIC is expressed at high levels in liver cancer cells and tumor tissues and is associated with a poor prognosis.** (**A**) Survival curve of patients with high and low ATIC expression (TCGA database). (**B**) ATIC expression in different cell lines. (**C**) ATIC mRNA levels in tumor tissues and adjacent normal tissues (patients treated at Zhongnan Hospital). (**D**) ATIC mRNA expression in cells transfected with the siRNA. (**E**) ATIC protein expression level in tumor tissues and adjacent normal tissues. (**F**) ATIC protein expression in different cell lines. (**G**) Protein expression level after ATIC knockdown. NC, negative control. ns, not significant.

Since the hazard ratio of ATIC was greater than that of other genes and it had a large coefficient in the established model, we decided to functionally verify the effects of ATIC. According to follow-up and gene expression data from TCGA, high ATIC expression was closely related to a poor prognosis (P<0.001, Fig. 5A). Compared with the normal liver cell line LO2, the HepG2 and Huh7 cell lines showed significantly higher expression of ATIC (Fig. 5B). We collected tumor samples and paracancerous samples from 52 patients with liver cancer from the Biological Repositories, Zhongnan Hospital of Wuhan University and verified that ATIC was expressed at significantly higher levels in liver cancer tissue samples (P<0.001, Fig. 5C).

High levels of ATIC-associated aggressive liver cancer pathology will lead to a poor prognosis in terms of patient survival. We analyzed the association between ATIC and the clinicopathological characteristics of patients with liver cancer. As shown in Table 2, ATIC expression was associated with lymph node invasion (P = 0.048) and the prognosis (Fig. S1). However, ATIC expression was not associated with age (P = 0.393), sex (P = 0.760) and portal vein tumor thrombosis (PVTT, P = 0.405) (Table 2). We also analyzed the relative risk of ATIC in the prognosis of liver cancer. The results of the Cox regression analysis showed that, compared with patients with liver cancer in the low ATIC expression group, high ATIC expression was closely related to a poor prognosis of patients with liver cancer (P=0.002). The multivariate Cox regression analysis further confirmed that ATIC expression was significantly correlated with the prognosis of patients with liver cancer (P=0.006) (Table 3).

We used HepG2 and Huh7 cell lines for the next experiment. After transfection, expression levels of the ATIC mRNA and protein were significantly decreased (Fig. 5D). The western blot results were consistent with the PCR results, showing that ATIC was expressed at high levels in liver cancer tissues (Fig. 5E) and liver cancer cell lines (Fig. 5F). The siRNA reduced the level of the ATIC mRNA and protein (Fig. 5G).

### Knockdown of ATIC promotes autophagy and apoptosis and inhibits malignant tumor behaviors *in vitro*

**Figure 6:**
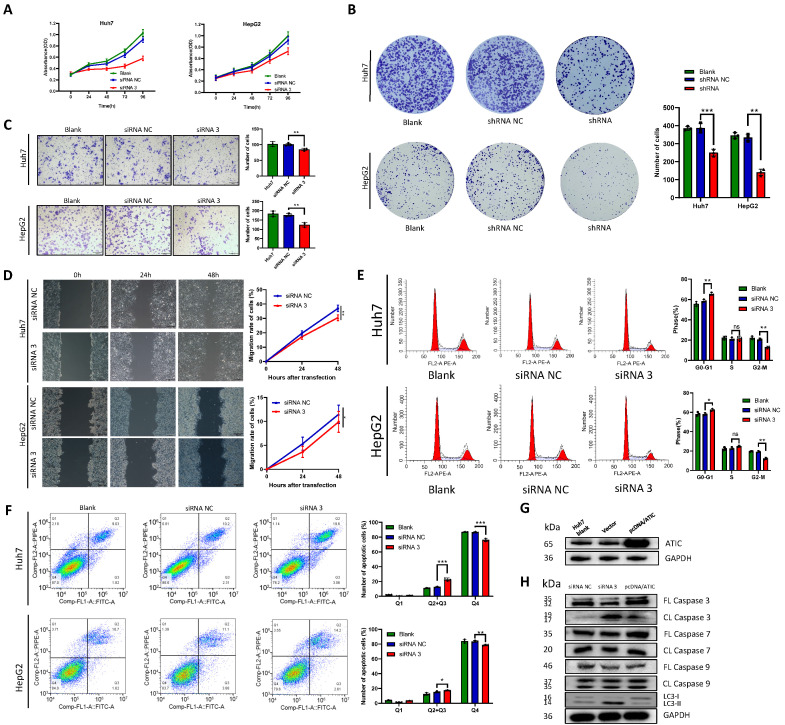
** ATIC knockdown inhibits the malignant behavior of tumor cells.** (**A**) Cell viability detected using CCK-8 assays. (**B**) Representative micrographs and colony numbers from the colony formation assays. (**C**) Representative micrographs and cell numbers from the Transwell assays. (**D**) Wound healing assay results showing the differences in migratory capacities (left panel); statistical analysis of the wound healing assay results (right panel). (**E**) Cell cycle assays conducted using flow cytometry. (**F**) Apoptosis assays conducted using flow cytometry. (**G**) ATIC protein expression after transfection of pcDNA/ATIC. (**H**) Apoptosis and autophagy-related protein expression after transfection of the siRNA. FL caspase, full-length caspase; CL caspase, cleaved caspase. *, P<0.05; **, P<0.01; and ***, P<0.001. ns, not significant.

The negative control siRNA (siRNA NC) and siRNA 3 were transfected into the liver cancer cell lines Huh7 and HepG2, and the CCK-8 assay was used to detect cell proliferation. After ATIC knockdown, cell proliferation decreased significantly (P<0.05, Fig. 6A). Liver cancer cells were transfected with shRNA NC and the ATIC shRNA. The results of the colony formation experiment showed that after 2 weeks of culture, the number of colonies formed by the cells with ATIC knockdown was significantly lower than the number of colonies formed by control cells (P<0.05, Fig. 6B). Transwell and wound healing experiments showed that ATIC knockdown reduced the invasion and migration of cells (Fig. 6C and D). Consistent with the results of the enrichment analysis, ATIC knockdown affected the cell cycle of tumor cells (Fig. 6E). Apoptosis and autophagy are closely related. ATIC knockdown induced cell apoptosis (Fig. 6F). After the transfection of pcDNA/ATIC, the level of ATIC protein increased significantly (Fig. 6G). The levels of LC3 I and LC3 II reflect cell autophagy. An increase in the proportion of LC3 II represents an increase in the level of autophagy. After ATIC knockdown, the levels of cleaved caspase 3 in cells increased, and at the same time, the proportion of the autophagy-related protein LC3 II increased (Fig. 6H). The levels of cleaved caspase 7 and 9 did not change, indicating that ATIC not only alters the level of autophagy but also affects the caspase 3-related apoptosis pathway.

### ATIC knockdown inhibits tumor growth and metastasis *in vivo*, and overexpression of ATIC promotes the proliferation and migration of tumor cells

**Figure 7:**
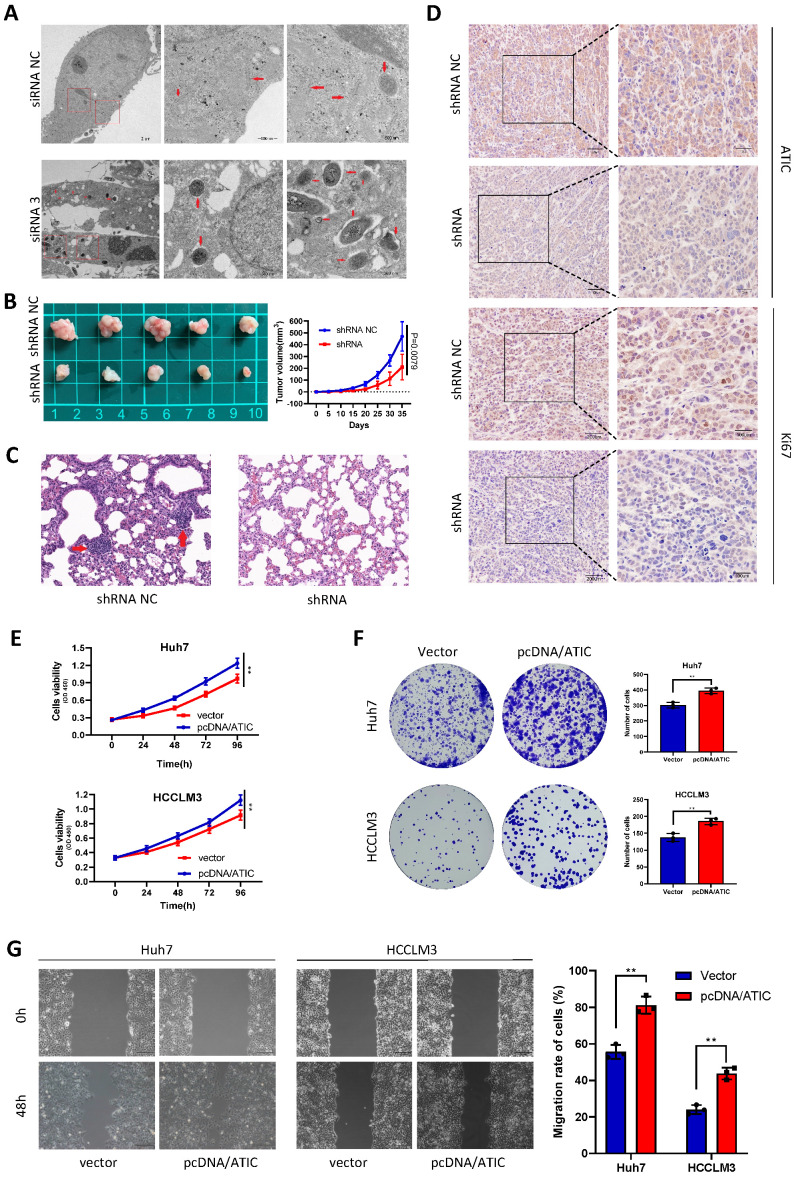
** ATIC inhibits autophagy and affects the malignant behavior of tumors *in vivo* and *in vitro*.** (**A**) Autophagosomes observed using electron microscopy. (**B**) Statistical analysis of tumor volumes in the two groups. (**C**) Representative images of lung metastases observed in the two groups of nude mice. (**D**) H&E staining of xenograft tumors. (**E and F**) ATIC overexpression promoted tumor proliferation. (**G**) ATIC overexpression promoted tumor migration, as assessed using wound healing assays. *, P<0.05; **, P<0.01; and ***, P<0.001.

After knocking down ATIC in Huh7 cells, intracellular autophagosomes (shown by the red arrow) were observed under a transmission electron microscope. The number of intracellular autophagosomes was significantly increased after siRNA treatment, consistent with the changes in LC3 levels (Fig. 7A). In the transplanted tumor mouse model, the growth rate of tumor cells with ATIC knockdown was significantly slower than that of control cells, and the tumor volume was significantly smaller than that of the control group (Fig. 7B). The lung metastasis ability of tumor cells was significantly reduced after ATIC knockdown (Fig. 7C). The immunohistochemistry results showed lower expression of the ATIC and Ki67 proteins in tumors treated with the shRNA than in control tumors (Fig. 7D). After the transfection of pcDNA/ATIC, the proliferation and migration of tumor cells were significantly increased (Fig. 7E-G).

### ATIC inhibits autophagy through the AKT/FOXO3 pathway

**Figure 8:**
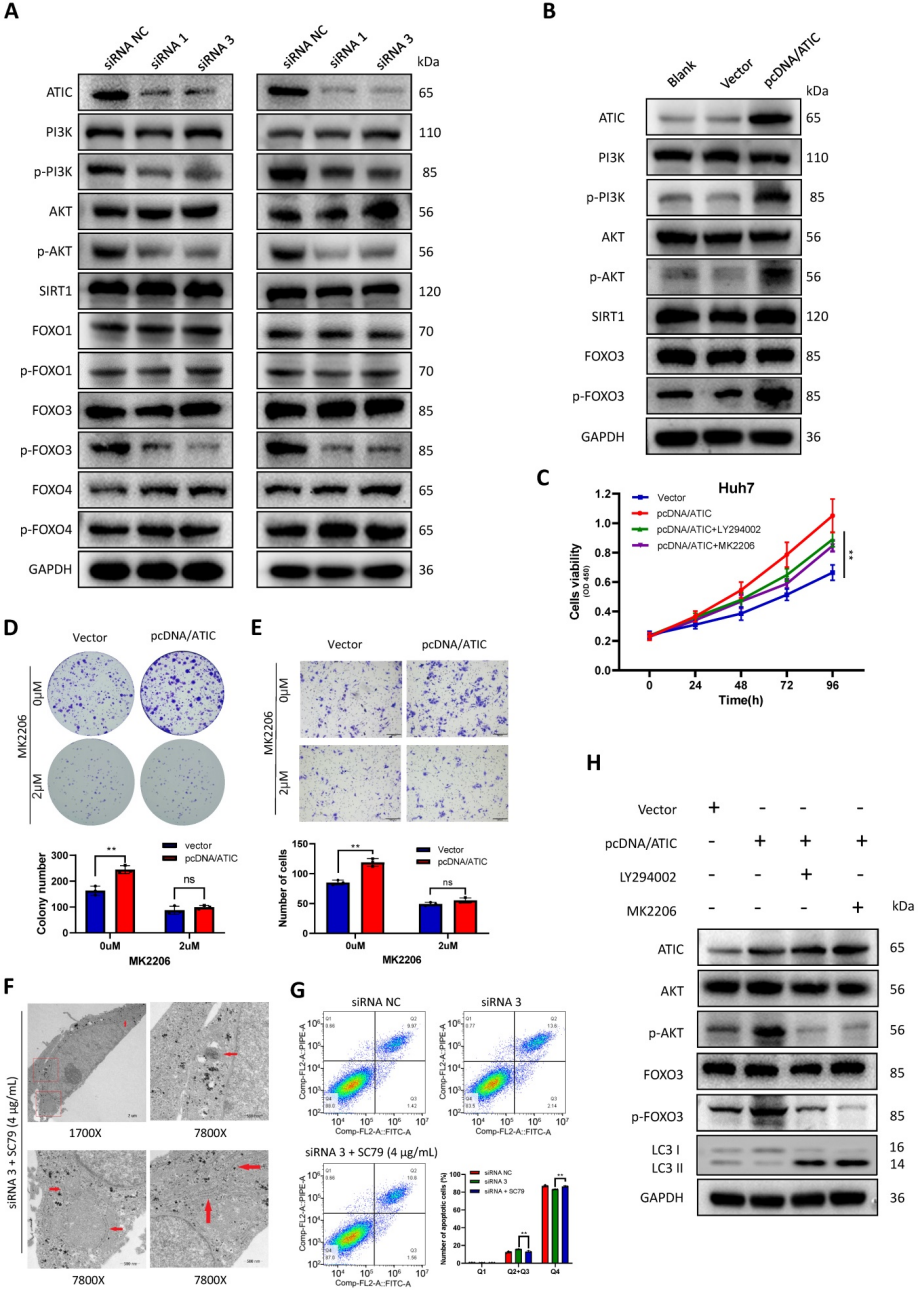
** ATIC inhibits autophagy through the AKT/FOXO3 pathway.** (**A**) Western blot analysis of the AKT/FOXO3 pathway after ATIC knockdown. (**B**) Western blot analysis of the AKT/FOXO3 pathway after ATIC overexpression. (**C**) Cell viability assays after exposure to different treatments. (**D**) Colony formation assays with the indicated cells. (**E**) Transwell results. (**F**) Electron microscopy images of cells treated with siRNA 3 and SC79. (**G**) Apoptosis assay conducted using flow cytometry. (**H**) Western blot analysis of the AKT/FOXO3 pathway and LC3 levels in different treated cells. ns, not significant.

KEGG (www.genome.jp/kegg/) pathway analysis showed that ATIC was associated with metabolic pathways, the cell cycle and the FOXO signaling pathway (Table S3). The literature suggests a close association between the FOXO pathway and autophagy 15, 16; therefore, we tested the FOXO pathway after knocking down ATIC. FOXO3 induces autophagy by upregulating multiple autophagy-related genes 17. However, the phosphorylation of FOXO3 results in its export from the nucleus and subsequent degradation. Therefore, phosphorylation of FOXO3 suppresses autophagy 18. After knocking down ATIC, FOXO3 phosphorylation decreased, while the phosphorylation of FOXO1 and FOXO4 was not affected by ATIC knockdown. Because FOXO3 is regulated by SIRT1 or PI3K/AKT under certain conditions 19-21, we examined SIRT1 and PI3K/AKT activity. The FOXO3 phosphorylation observed in this study was mediated by activation of the PI3K/AKT pathway rather than SIRT1 (Fig. 8A). Overexpression of ATIC resulted in PI3K-induced AKT activation and FOXO3 hyperphosphorylation (Fig. 8B). The PI3K/AKT pathway inhibitors LY294002 and MK2206 were used for rescue experiments. LY294002 (50 uM) and MK2206 (2 uM) reversed the increase in cell proliferation and migration caused by ATIC overexpression (Fig. 8 C-E). When cells were simultaneously treated with the siRNA and SC79, the number of autophagosomes observed under the transmission electron microscope was significantly reduced compared with siRNA-treated cells (Fig. 8F). Apoptosis induced by ATIC knockdown was also reversed by SC79 (Fig. 8G). After adding LY294002 or MK2206, the increase in p-AKT and p-FOXO3 levels caused by pcDNA/ATIC treatment was reversed. Similarly, the use of these inhibitors reversed the changes in LC3 protein levels caused by ATIC overexpression (Fig. 8H).

## Discussion

Given the heterogeneous nature of HCC, a satisfactory risk assessment and clinical management of patients with liver cancer are difficult 22, 23. Therefore, we want to establish a prognostic model to evaluate the risk and survival of patients and to provide guidance for clinical treatment.

Autophagy is regulated by autophagy-related genes, and the abnormal expression of autophagy-related genes affects the occurrence and development of tumors by modulating autophagy 24. Due to the relationship between autophagy-related gene expression and tumor progression, establishing a liver cancer prognostic model based on autophagy-related genes is reasonable. The prognostic model constructed in this study can predict whether a patient is at high risk of a poor prognosis and the long-term survival rate of the patient. Based on the results of GO and KEGG analyses, autophagy-related genes were closely related to mitosis and the cell cycle. Consistent with this finding, ATIC is known to affect the cell cycle and promote cell proliferation.

Autophagy-related genes inhibit tumor growth by affecting immune cell infiltration, but few studies have assessed the relationship between autophagy and immune cell infiltration 25. Results of the immune cell infiltration analysis suggest that the autophagy-related genes in the model are related to the tumor immune microenvironment, the activity of immune cells, and immune function, but the mechanisms underlying these relationships have not yet been completely elucidated. HDAC1 has been reported to affect antigen presentation and immune activation. Therefore, the relationship between autophagy-related genes such as ATIC, PRKCD and SPNS1 and immune function deserves further study 26. We grouped patients according to the expression of ATIC and analyzed the difference in immune cell infiltration between the two groups. CD8+ T cells, macrophages, immune checkpoints and IFN immune responses were different between the two groups. The main immune cell related to immunotherapy is CD8+ T cells 27, 28; thus, we speculate that ATIC is related to the tumor immune microenvironment and immunotherapy. Autophagy modulates immunity through many mechanisms, including abnormal autophagy interfering with the survival and activity of T cells or autophagy activation promoting and inhibiting the secretion of cytokines 29, 30. These findings suggest that the induction or suppression of autophagy combined with immunotherapy may be a prospective treatment strategy 31.

We screened small-molecule drugs with therapeutic potential based on changes in the gene expression levels observed in patients in the high-risk group, but *in vivo* and *in vitro* experimental verification was not performed. Changes in the gene expression profile of cells treated with these small-molecule drugs were exactly the opposite of the gene expression profile of patients in the high-risk group. Thus, these small-molecule drugs exert a potential therapeutic effect on liver cancer. Among them, doxorubicin has been widely used as a clinical treatment for liver cancer 32. Icariin was reported to inhibit tumor cell proliferation, but it has not been studied in liver cancer 33, 34. We will conduct research on the treatment effects and resistance of the other molecules in the future.

ATIC is related to tumor cell proliferation, rheumatoid arthritis and the efficacy of radiotherapy 35, 36. No previous report has documented the correlations between ATIC and autophagy and the tumor immune microenvironment. In this study, we proved through *in vivo* and *in vitro* experiments that ATIC promotes the progression of liver cancer through the AKT/FOXO3 pathway. Our model is related to patient prognosis and immune cell infiltration, and it has been verified using external databases, which further proves the reliable prognostic ability of our model. Previous studies have shown that autophagy is related to immune cell infiltration and tumor immune tolerance 37, 38. We found that ATIC, an autophagy-related gene, is related to immune cell infiltration by performing a bioinformatics analysis, but the specific mechanism remains unclear. We selected ATIC for verification in this study, but in subsequent studies, we will also verify the relationship between several other genes and the immune microenvironment and conduct in-depth research on the mechanism by which ATIC alters immune responses.

In the analysis of the correlation between ATIC expression and the clinicopathological characteristics of patients with liver cancer, we found that ATIC expression is related to lymph node invasion and patient prognosis. The TNM stage of the high ATIC expression group was higher, but the difference was not significant. We propose that this result may be attributed to our small sample size, and we will include more patients in future studies.

The prognostic model we established can effectively predict the prognostic risk of patients and provide guidance for clinical decision-making. For example, after a patient has undergone liver cancer resection, are adjuvant treatments such as transcatheter arterial chemoembolization (TACE) necessary 39? If the patient is a high-risk patient based on the model score, other adjuvant treatments are also recommended, even if the tumor is a well-differentiated liver cancer.

At the same time, we screened many genes with research value by performing a bioinformatics analysis. For example, HDAC1 is closely related to the prognosis of patients with liver cancer according to the results of the multivariate Cox regression analysis, but a report describing the mechanism by which it promotes tumor occurrence and tumor development has not been published. Therefore, we hope that the results of our analysis provide ideas for future research on the role of autophagy in tumors.

The constructed prognostic model is potentially useful to predict the survival rate of patients, and the predictive performance has been verified in an external ICGC dataset to support its validity. We verified that the key gene ATIC affects tumor progression by modulating autophagy *in vitro* and *in vivo*, and the effect of ATIC on autophagy in liver cancer has been clarified here for the first time.

## Supplementary Material

Supplementary figures and tables 1 and 3.

Supplementary table 2: Results of cMAP.

## Figures and Tables

**Table 1:** Correlation between the risk score and clinicopathological characteristics of patients from TCGA and ICGC databases

Characteristic	TCGA cohort			ICGC cohort		
	High risk	Low risk	χ^2^	High risk	Low risk	χ^2^
No. of patients	182	183		115	116	
**Age**			0.120			0.061
≥65 y	67	82		81	68	
<65 y	115	101		34	48	
**Sex**			0.882			0.480
Male	122	124		87	83	
Female	60	59		28	33	
**Stage**			0.010			0.006
I	69	101		13	23	
II	48	36		45	60	
III	51	32		43	28	
IV	1	3		14	5	
Unknown	13	11		0	0	
**T**			0.004			
T1	74	106		NA	NA	
T2	54	37		NA	NA	
T3	45	33		NA	NA	
T4	9	4		NA	NA	
Unknown	0	3		NA	NA	
**N**			0.615			
N0	128	120		NA	NA	
N1	2	2		NA	NA	
Unknown	52	61		NA	NA	
**M**			0.712			
M0	134	129		NA	NA	
M1	1	2		NA	NA	
Unknown	47	52		NA	NA	
**Grade**			<0.001			
Grade 1	17	38		NA	NA	
Grade 2	77	98		NA	NA	
Grade 3	76	42		NA	NA	
Grade 4	10	2		NA	NA	
Unknown	2	3		NA	NA	

**Table 2:** Correlation between ATIC expression and the clinicopathological characteristics of patients with liver cancer

Characteristics	ATIC		P/χ^2^ value
	Low	High	
Age (y)	62.12±10.667	59.77±8.887	0.393
**Sex**			0.760
**Male**	19	18	
Female	7	8	
**HBV infection**			0.734
Yes	21	20	
No	5	6	
**AFP**			0.780
≥400	15	14	
<400	11	12	
Tumor diameter (cm)	3.819±1.828	4.735±1.617	0.062
**TNM classification**			0.061
I	2	0	
II	13	7	
III	8	13	
IV	3	6	
**PVTT**			0.405
Yes	12	15	
No	14	11	
**Lymphatic invasion**			0.048
Yes	7	14	
No	19	12	

PVTT, portal vein tumor thrombosis; HBV, hepatitis B virus; AFP, alpha-fetoprotein.

**Table 3:** Univariate and multivariate Cox regression analyses of various prognostic parameters in patients with liver cancer

	Univariate analysis			Multivariate analysis		
	P	HR	95% CI	P	HR	95% CI
Age	0.345	1.020	0.979-1.062			
Sex (male)	0.590	0.798	0.351-1.814			
HBV	0.412	1.432	0.608-3.371			
TNM classification	<0.001	7.021	3.686-13.372	0.001	4.407	1.870-10.383
Tumor diameter	0.003	1.386	1.115-1.721			
PVTT	0.001	4.286	1.798-10.217	0.006	5.969	1.686-21.128
Lymphatic invasion	0.068	0.501	0.238-1.052			
AFP	0.001	5.048	1.990-12.807	0.029	4.297	1.157-15.967
ATIC	0.002	1.593	1.186-2.139	0.006	2.099	1.243-3.544

HR, Hazard ratio; CI, confidence interval.

## Abbreviations

ATIC: 5-aminoimidazole-4-carboxamide ribonucleotide formyltransferase/inosine monophosphate (IMP) cyclohydrolase
TCGA: The Cancer Genome Atlas
ICGC: International Cancer Genome Consortium
logFC: log_2_ fold change
FDR: false discovery rate
CCK-8: cell counting kit 8
AUC: area under the curve
siRNA NC: siRNA negative control
