# Supplementary material for: ATIC inhibits autophagy in hepatocellular cancer through the AKT/FOXO3 pathway and serves as a prognostic signature for modeling patient survival
Source: Int J Biol Sci. 2021 Oct 25;17(15):4442–58. doi: 10.7150/ijbs.65669 (PMC8579461; doi:10.7150/ijbs.65669)
Supplement: Supplementary file 1 — Supplementary figures and tables 1 and 3. [file ijbsv17p4442s1.pdf]

Fig. S1 survival curve of 52 patients

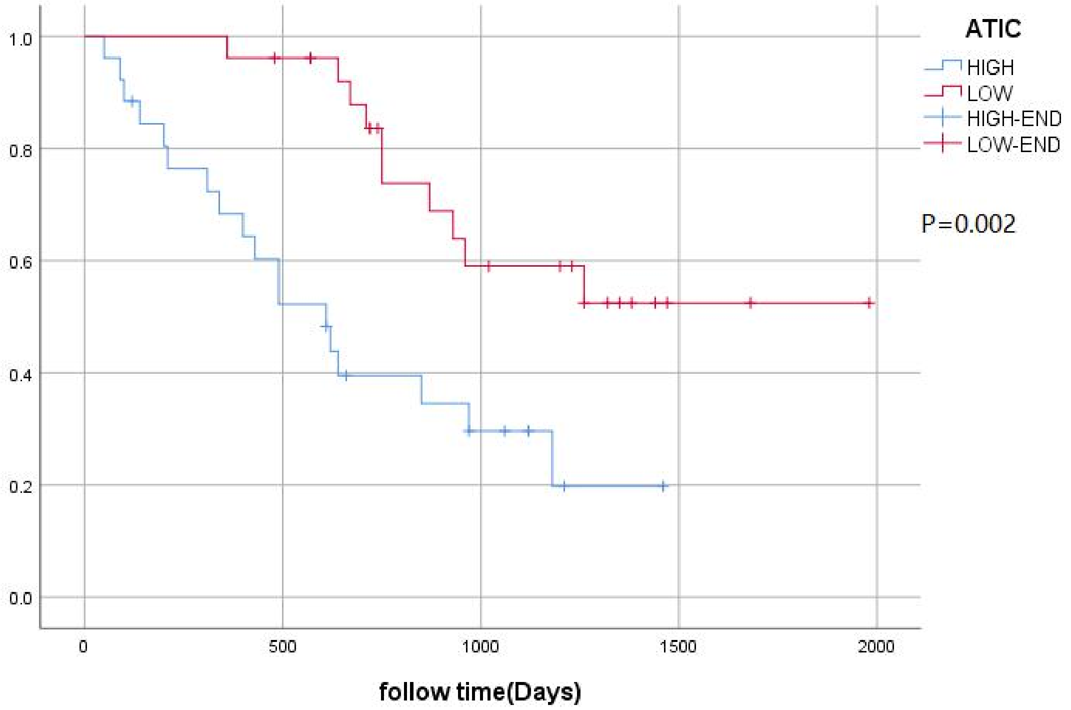

Table S1 23 overlapping genes

Gene symbol	Mean Exp in normal	Mean Exp in Tumor	logFC	P-Value	FDR
BIRC5	0.251	7.050	4.815	2.35E-28	4.56E-26
PEA15	12.163	43.368	1.834	3.57E-27	3.46E-25
RAB24	1.365	4.436	1.701	8.37E-27	4.06E-25
HSP90AB1	114.178	354.407	1.634	3.53E-26	1.37E-24
CAPN10	0.580	1.639	1.499	4.96E-26	1.59E-24
MAPK3	3.657	9.508	1.379	5.73E-26	1.59E-24
CDKN2A	0.163	4.098	4.654	1.87E-25	4.53E-24
RHEB	6.866	15.941	1.215	2.98E-25	6.41E-24
ATG4B	3.061	6.377	1.059	4.52E-24	7.31E-23
ATIC	7.758	18.695	1.269	1.56E-23	2.21E-22
FKBP1A	22.160	49.666	1.164	2.89E-23	3.38E-22
ATG10	0.644	1.308	1.023	1.11E-22	8.98E-22
NPC1	1.411	3.747	1.409	1.77E-20	9.80E-20
GAPDH	278.515	661.696	1.248	5.02E-20	2.63E-19
HDAC1	9.673	19.697	1.026	6.77E-19	2.92E-18
BAK1	2.433	7.457	1.616	1.55E-17	6.01E-17
SQSTM1	29.987	99.918	1.736	9.02E-17	3.02E-16
PRKCD	2.370	6.312	1.413	8.94E-16	2.94E-15
CASP8	1.377	2.831	1.040	1.52E-15	4.83E-15
SPNS1	0.255	0.636	1.316	2.15E-14	6.14E-14
IKBKE	0.548	2.003	1.869	1.30E-11	3.00E-11
TMEM74	0.125	0.630	2.334	3.59E-11	8.00E-11
RGS19	2.019	4.376	1.116	5.15E-10	1.04E-09

Table S3 KEGG result of ATIC

Category	Term	Count
KEGG_PATHWAY	hsa03010:Ribosome	110
KEGG_PATHWAY	hsa03008:Ribosome biogenesis in eukaryotes	49
KEGG_PATHWAY	hsa01100:Metabolic pathways	416
KEGG_PATHWAY	hsa03040:Spliceosome	65
KEGG_PATHWAY	hsa03013:RNA transport	76
KEGG_PATHWAY	hsa04110:Cell cycle	58
KEGG_PATHWAY	hsa00983:Drug metabolism - other enzymes	27
KEGG_PATHWAY	hsa00240:Pyrimidine metabolism	47
KEGG_PATHWAY	hsa00280:Valine, leucine and isoleucine degradat	26
KEGG_PATHWAY	hsa04919:Thyroid hormone signaling pathway	51
KEGG_PATHWAY	hsa01130:Biosynthesis of antibiotics	84
KEGG_PATHWAY	hsa04922:Glucagon signaling pathway	45
KEGG_PATHWAY	hsa04610:Complement and coagulation cascades	34
KEGG_PATHWAY	hsa04068:FoxO signaling pathway	57
KEGG_PATHWAY	hsa00071:Fatty acid degradation	23
KEGG_PATHWAY	hsa04931:Insulin resistance	47
KEGG_PATHWAY	hsa04146:Peroxisome	38
KEGG_PATHWAY	hsa00650:Butanoate metabolism	16
KEGG_PATHWAY	hsa05130:Pathogenic Escherichia coli infection	25
KEGG_PATHWAY	hsa04114:Oocyte meiosis	46
KEGG_PATHWAY	hsa04910:Insulin signaling pathway	55
KEGG_PATHWAY	hsa04710:Circadian rhythm	17
KEGG_PATHWAY	hsa00260:Glycine, serine and threonine metabolis	20
KEGG_PATHWAY	hsa03018:RNA degradation	33
KEGG_PATHWAY	hsa01200:Carbon metabolism	45
KEGG_PATHWAY	hsa00250:Alanine, aspartate and glutamate metal	18
KEGG_PATHWAY	hsa04976:Bile secretion	30
KEGG_PATHWAY	hsa00310:Lysine degradation	24
KEGG_PATHWAY	hsa04152:AMPK signaling pathway	48
KEGG_PATHWAY	hsa04914:Progesterone-mediated oocyte matura	36
KEGG_PATHWAY	hsa03050:Proteasome	21
KEGG_PATHWAY	hsa00410:beta-Alanine metabolism	16
KEGG_PATHWAY	hsa04141:Protein processing in endoplasmic retic	62
KEGG_PATHWAY	hsa04932:Non-alcoholic fatty liver disease (NAFL	56
KEGG_PATHWAY	hsa00380:Tryptophan metabolism	19
KEGG_PATHWAY	hsa01230:Biosynthesis of amino acids	30
KEGG_PATHWAY	hsa05213:Endometrial cancer	23
KEGG_PATHWAY	hsa00350:Tyrosine metabolism	17
KEGG_PATHWAY	hsa05215:Prostate cancer	35
KEGG_PATHWAY	hsa04920:Adipocytokine signaling pathway	29
KEGG_PATHWAY	hsa05219:Bladder cancer	19
KEGG_PATHWAY	hsa00120:Primary bile acid biosynthesis	10
KEGG_PATHWAY	hsa00360:Phenylalanine metabolism	10
KEGG_PATHWAY	hsa00220:Arginine biosynthesis	11
KEGG_PATHWAY	hsa03060:Protein export	12
KEGG_PATHWAY	hsa03320:PPAR signaling pathway	27
KEGG_PATHWAY	hsa05230:Central carbon metabolism in cancer	26
KEGG_PATHWAY	hsa00030:Pentose phosphate pathway	14
KEGG_PATHWAY	hsa04915:Estrogen signaling pathway	37
KEGG_PATHWAY	hsa03450:Non-homologous end-joining	8
KEGG_PATHWAY	hsa04923:Regulation of lipolysis in adipocytes	23
KEGG_PATHWAY	hsa00630:Glyoxylate and dicarboxylate metabolis	13
KEGG_PATHWAY	hsa03030:DNA replication	16
KEGG_PATHWAY	hsa04722:Neurotrophin signaling pathway	43
KEGG_PATHWAY	hsa04130:SNARE interactions in vesicular transpo	15
KEGG_PATHWAY	hsa04520:Adherens junction	27
KEGG_PATHWAY	hsa05100:Bacterial invasion of epithelial cells	29

KEGG_PATHWAY	hsa00330:Arginine and proline metabolism	20
KEGG_PATHWAY	hsa00230:Purine metabolism	59

%	PValue	Genes	List Total
2.158556	4.32E-37	RPL4, RPL5, RPL30, RPL3, RPL32, RPL31, RPL34, RPL10A, RPL8	1935
0.961538	8.90E-08	POP5, RBM28, POP7, NXT1, POP1, NVL, POP4, RPP30, NAT1(	1935
8.163265	3.26E-07	PNMT, PI4K2B, CDA, GLDC, UXS1, ABAT, ENO1, IL4I1, SCP2, T	1935
1.27551	6.16E-07	ISY1, EIF4A3, HNRNPU, PRPF19, USP39, PQBP1, EFTUD2, SAR	1935
1.491366	7.80E-06	POP5, EIF4A1, POP7, NXT1, POP1, POP4, FMR1, RPP30, EIF4A	1935
1.138148	1.47E-05	YWHAE, GSK3B, CDKN1A, CDKN1B, MCM7, BUB1B, CDC14B,	1935
0.529827	4.69E-05	CDA, TPMT, DPYS, GMPS, CYP3A4, UGT1A3, UPP2, TK2, UPP1	1935
0.922292	1.27E-04	CDA, DTYMK, CANT1, DPYS, NUDT2, NME1-NME2, NT5C, PC	1935
0.510204	2.43E-04	ACAA2, HIBADH, ABAT, AACS, ACAT1, IL4I1, MCEE, ALDH2, D	1935
1.000785	2.70E-04	GSK3B, THRB, THRA, ITGB3, SLC2A1, ACTB, ACTG1, MED14, M	1935
1.648352	2.77E-04	ACAA2, GLDC, TAT, OGDHL, ENO1, FNTA, ALDH2, DBT, ACA	1935
0.883046	3.45E-04	CAMK2B, PFKFB1, CRTC2, PRKAA1, PRKAA2, PDE3B, SLC2A1,	1935
0.66719	3.72E-04	CPB2, CFH, C1S, C1R, SERPINC1, PROS1, CFI, C4BPA, PLG, C8	1935
1.118524	4.13E-04	CDKN1A, CDKN1B, IRS1, SETD7, PTEN, PRKAG2, IRS2, AKT2, T	1935
0.451334	7.51E-04	GCDH, CPT1A, ACAA2, ADH1C, ACSL1, ADH1B, ADH1A, ECI2	1935
0.922292	8.07E-04	GSK3B, CRTC2, PRKAA1, PRKAA2, IRS1, PTEN, SLC2A1, PRKA(	1935
0.745683	9.29E-04	ABCD3, ECI2, HSD17B4, PRDX5, SCP2, PRDX1, HAO1, MPV17	1935
0.313972	0.002442	ACSM3, ACSM2A, ACSM5, ABAT, AACS, ACSM2B, ACAT1, BD	1935
0.490581	0.002981	ROCK1, ARPC1B, ARPC1A, ARPC5L, WASL, ACTB, ACTG1, TUI	1935
0.902669	0.003013	YWHAE, CAMK2B, ADCY4, ITPR1, ITPR2, CALML3, ADCY1, PK	1935
1.079278	0.003097	GSK3B, IRS1, PDE3B, PRKAG2, CALML3, IRS2, PYGL, GYS2, LIP	1935
0.333595	0.004727	PRKAA1, PRKAA2, RORC, PRKAG2, RORA, CSNK1E, ARNTL, R	1935
0.392465	0.004928	DMGDH, AOC3, SDS, MAOB, MAOA, AGXT2, GLDC, SHMT1,	1935
0.647567	0.007411	ZCCHC7, DIS3L, ENO1, TOB2, TOB1, HSPD1, EDC3, EXOSC7,	1935
0.883046	0.007906	H6PD, GLDC, SHMT1, RPE, OGDHL, GPT, ENO1, ACAT1, MCE	1935
0.353218	0.007959	ADSL, AGXT2, GLS2, GPT2, CAD, GOT2, ASNS, ABAT, GPT, AS	1935
0.588697	0.008763	AQP8, ABCB4, AQP9, SLC22A1, ADCY4, SLC2A1, AQP4, ADCY	1935
0.470958	0.009176	KMT2E, SUV39H2, GCDH, SETD2, PHYKPL, AADAT, EHMT2, K	1935
0.941915	0.009239	PFKFB1, PFKFB4, CRTC2, PRKAA1, PRKAA2, IRS1, PRKAG2, PIK	1935
0.706436	0.009521	HSP90AB1, PDE3B, ADCY4, PIK3R3, PIK3R2, ADCY1, PIK3R1, F	1935
0.412088	0.010309	PSMD14, PSMD13, POMP, PSMA7, PSMD8, PSMA5, PSMB6, I	1935
0.313972	0.012918	AOC3, SMOX, DPYS, ABAT, UPB1, CNDP1, SRM, ALDH6A1, A	1935
1.216641	0.013613	ERO1B, HSP90AB1, UBE2D2, MAN1A2, SEC61G, MAN1A1, SE	1935
1.098901	0.015259	GSK3B, NDUFA13, GSK3A, NDUFA12, IRS1, NDUFA10, PRKAG	1935
0.372841	0.016414	GCDH, MAOB, MAOA, AADAT, HAAO, OGDHL, KMO, ACAT1	1935
0.588697	0.016982	SHMT1, RPE, TAT, GPT, ENO1, CBS, PSPH, SDS, TPI1, ARG1, G	1935
0.451334	0.019211	GSK3B, MAP2K1, MAP2K2, PDPK1, LEF1, PTEN, PIK3R3, ILK, P	1935
0.333595	0.019524	PNMT, AOC3, MAOB, HGD, ADH1C, MAOA, ADH1B, TAT, AD	1935
0.686813	0.020481	GSK3B, CDKN1A, CDKN1B, HSP90AB1, LEF1, PTEN, PIK3R3, P	1935
0.569074	0.020743	PRKAA1, PRKAA2, IRS1, SLC2A1, PRKAG2, IRS2, ACACB, ADIP	1935
0.372841	0.021744	CDKN1A, MAP2K1, MAP2K2, DAPK1, MMP1, SRC, DAPK3, MI	1935
0.196232	0.026641	CYP39A1, ACOX2, SCP2, AKR1D1, HSD17B4, BAAT, CYP46A1	1935
0.196232	0.026641	AOC3, IL4I1, MAOB, MAOA, TAT, PAH, GOT2, GLYAT, MIF, HI	1935
0.215856	0.031114	NOS2, CPS1, GLS2, NOS3, ARG1, GPT2, GOT2, GPT, NAGS, A	1935
0.235479	0.034756	IMMP1L, SRP19, SPCS3, SPCS1, SEC61G, SRP54, SRPRA, SRPR	1935
0.529827	0.037277	ILK, CYP7A1, RXRA, SCP2, CPT2, ACADL, ACADM, PCK1, PCK2	1935
0.510204	0.037487	GLS2, PTEN, SLC2A1, SLC2A2, PIK3R3, PIK3R2, SLC1A5, PIK3R	1935
0.274725	0.040022	G6PD, H6PD, RPE, TALDO1, PGD, DERA, RPIA, RGN, PGLS, AL	1935
0.72606	0.044584	SHC4, HSP90AB1, SHC1, SRC, ADCY4, ITPR1, PIK3R3, ITPR2, (	1935
0.156986	0.044824	XRCC6, FEN1, DCLRE1C, XRCC4, XRCC5, LIG4, POLL, NHEJ1	1935
0.451334	0.046036	NPR1, IRS1, INSR, PTGER3, PDE3B, ADCY4, NPY1R, PIK3R3, PI	1935
0.255102	0.050957	GLDC, SHMT1, ACAT1, CS, GRHPR, MCEE, PCCA, CAT, PGP, A	1935
0.313972	0.055483	RNASEH2C, RNASEH2B, FEN1, RFC4, RNASEH2A, MCM7, RFC	1935
0.843799	0.058067	SHC4, YWHAE, CAMK2B, GSK3B, SHC1, IRS1, MAGED1, PIK3R	1935
0.294349	0.069385	STX17, STX8, GOSR1, STX18, STX10, VAMP8, STX1B, VAMP7,	1935
0.529827	0.073314	SRC, CTNND1, LEF1, PTPRJ, WASL, EGFR, ACTB, ACTG1, CDH	1935
0.569074	0.081339	SHC4, ARHGEF26, SHC1, SRC, ARPC1B, ARPC1A, ARPC5L, ILK	1935

0.392465	0.083005	MAOB, NOS2, MAOA, SMOX, NOS3, ARG1, GOT2, PYCR1, PY	1935
1.157771	0.088016	GDA, PDE3B, NUDT2, ENPP1, ENPP4, PDE8A, PGM1, ADSL, EI	1935

Pop Hits	Pop Total	Fold Enrich	Bonferroni	Benjamini	FDR
136	6879	2.875399	1.29E-34	1.29E-34	1.14E-34
87	6879	2.002263	2.65E-05	1.33E-05	1.18E-05
1219	6879	1.213204	9.70E-05	3.23E-05	2.88E-05
133	6879	1.737425	1.84E-04	4.59E-05	4.08E-05
172	6879	1.570831	0.002322	4.65E-04	4.14E-04
124	6879	1.662841	0.004366	7.29E-04	6.48E-04
46	6879	2.086653	0.01387	0.001995	0.001774
101	6879	1.654325	0.037014	0.004714	0.004192
47	6879	1.966617	0.06998	0.0075	0.006669
115	6879	1.576582	0.077431	0.0075	0.006669
212	6879	1.4086	0.079196	0.0075	0.006669
99	6879	1.615927	0.097836	0.008532	0.007587
69	6879	1.751758	0.105003	0.008532	0.007587
134	6879	1.512218	0.115737	0.008784	0.007811
42	6879	1.946807	0.200698	0.014929	0.013276
108	6879	1.5471	0.213847	0.015032	0.013367
83	6879	1.627608	0.241872	0.016281	0.014478
27	6879	2.10669	0.517454	0.040433	0.035955
51	6879	1.742666	0.589251	0.043942	0.039076
111	6879	1.473259	0.593098	0.043942	0.039076
138	6879	1.416863	0.60316	0.043942	0.039076
31	6879	1.949537	0.756329	0.063847	0.056776
39	6879	1.823097	0.770558	0.063847	0.056776
77	6879	1.523588	0.891039	0.09122	0.081119
113	6879	1.415723	0.906098	0.09122	0.081119
35	6879	1.828306	0.907562	0.09122	0.081119
69	6879	1.545669	0.927404	0.094577	0.084104
52	6879	1.640787	0.935876	0.094577	0.084104
123	6879	1.387332	0.937081	0.094577	0.084104
87	6879	1.471051	0.942208	0.094577	0.084104
44	6879	1.696723	0.954405	0.099097	0.088123
31	6879	1.834859	0.979237	0.120298	0.106976
169	6879	1.304215	0.983168	0.122927	0.109315
151	6879	1.318425	0.989767	0.133738	0.118928
40	6879	1.688643	0.992788	0.139755	0.124279
72	6879	1.481266	0.993928	0.140571	0.125004
52	6879	1.572421	0.996913	0.153112	0.136157
35	6879	1.726733	0.997193	0.153112	0.136157
88	6879	1.413936	0.997902	0.154539	0.137425
70	6879	1.472802	0.998063	0.154539	0.137425
41	6879	1.647457	0.998572	0.158044	0.140543
17	6879	2.091199	0.99968	0.184626	0.164181
17	6879	2.091199	0.99968	0.184626	0.164181
20	6879	1.955271	0.999919	0.210725	0.187389
23	6879	1.854803	0.999974	0.23016	0.204673
67	6879	1.432628	0.999988	0.23768	0.21136
64	6879	1.444234	0.999989	0.23768	0.21136
29	6879	1.716226	0.999995	0.248468	0.220953
99	6879	1.328651	0.999999	0.26715	0.237566
13	6879	2.187716	0.999999	0.26715	0.237566
56	6879	1.460105	0.999999	0.268997	0.239209
27	6879	1.711685	1	0.292023	0.259685
36	6879	1.580017	1	0.311962	0.277415
120	6879	1.273889	1	0.320444	0.284959
34	6879	1.568399	1	0.37594	0.334309
71	6879	1.351916	1	0.390135	0.346932
78	6879	1.321745	1	0.425246	0.378155

50	6879	1.422016	1	0.426475	0.379248
176	6879	1.191746	1	0.444557	0.395327
